# Clinical Presentation and Surgical Management of Idiopathic Gingival Fibromatosis: A Case Report

**DOI:** 10.7759/cureus.107138

**Published:** 2026-04-16

**Authors:** Racem Abid, Wafa Nasri, Ons Belgacem

**Affiliations:** 1 Periodontology, Oral Health, and Oro‐Facial Rehabilitation, Faculty of Dental Medicine of Monastir, University of Monastir, Monastir, TUN

**Keywords:** gingival fibromatosis, gingival overgrowth, gingivectomy, oral medicine and periodontology, periodontal surgeries

## Abstract

Idiopathic gingival fibromatosis (IGF) is a rare benign condition characterized by a progressive fibrous enlargement of the gingival tissues in the absence of systemic, pharmacological, or hereditary causes. This case report describes an 11-year-old male presenting with generalized gingival overgrowth causing functional and aesthetic impairment. Clinical examination revealed firm, pale pink, non-hemorrhagic gingival enlargement covering a significant portion of the dental crowns, with pseudo-pocket formation but no evidence of attachment loss. The patient had no relevant medical history, drug intake, or familial background suggestive of hereditary involvement.

Histopathological analysis demonstrated dense collagen bundles with elongated rete ridges and minimal inflammatory infiltrate, confirming the diagnosis of IGF. Following initial periodontal therapy to improve oral hygiene, surgical excision of the excess gingival tissue was performed. Postoperative healing was uneventful, and satisfactory functional and aesthetic outcomes were achieved. No recurrence was observed during the follow-up period of several months. Early diagnosis and appropriate surgical management combined with strict maintenance are essential to prevent recurrence and restore oral function in patients with IGF.

## Introduction

Gingival fibromatosis is a rare benign condition characterized by a slowly progressive enlargement of the gingival tissues resulting from excessive accumulation of fibrous connective tissue. The enlargement may be localized or generalized and is typically non-inflammatory in nature, presenting clinically as firm, pale pink gingiva with a dense fibrotic consistency. In severe cases, the overgrowth may partially or completely cover the dental crowns, leading to functional and aesthetic concerns [1].

Based on etiology, gingival fibromatosis can be classified as hereditary, idiopathic, or syndromic. Hereditary gingival fibromatosis is commonly transmitted in an autosomal dominant pattern and may present either as an isolated condition or as part of various genetic syndromes. In contrast, idiopathic gingival fibromatosis (IGF) occurs in the absence of identifiable genetic background, systemic disease, or drug-related causes and is considered a diagnosis of exclusion after eliminating inflammatory, pharmacologic, and systemic etiologies.

Clinically, IGF may interfere with tooth eruption, mastication, phonation, oral hygiene maintenance, and facial aesthetics, potentially affecting the patient’s psychological well-being. Management remains challenging due to the potential for recurrence following surgical excision and the need for meticulous long-term periodontal maintenance [1,2].

## Case presentation

**Figure 1:**
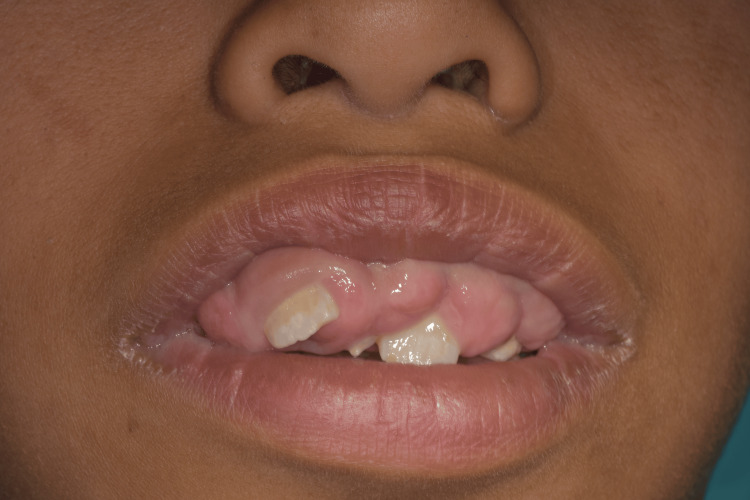
Extraoral view showing lip incompetence

**Figure 2:**
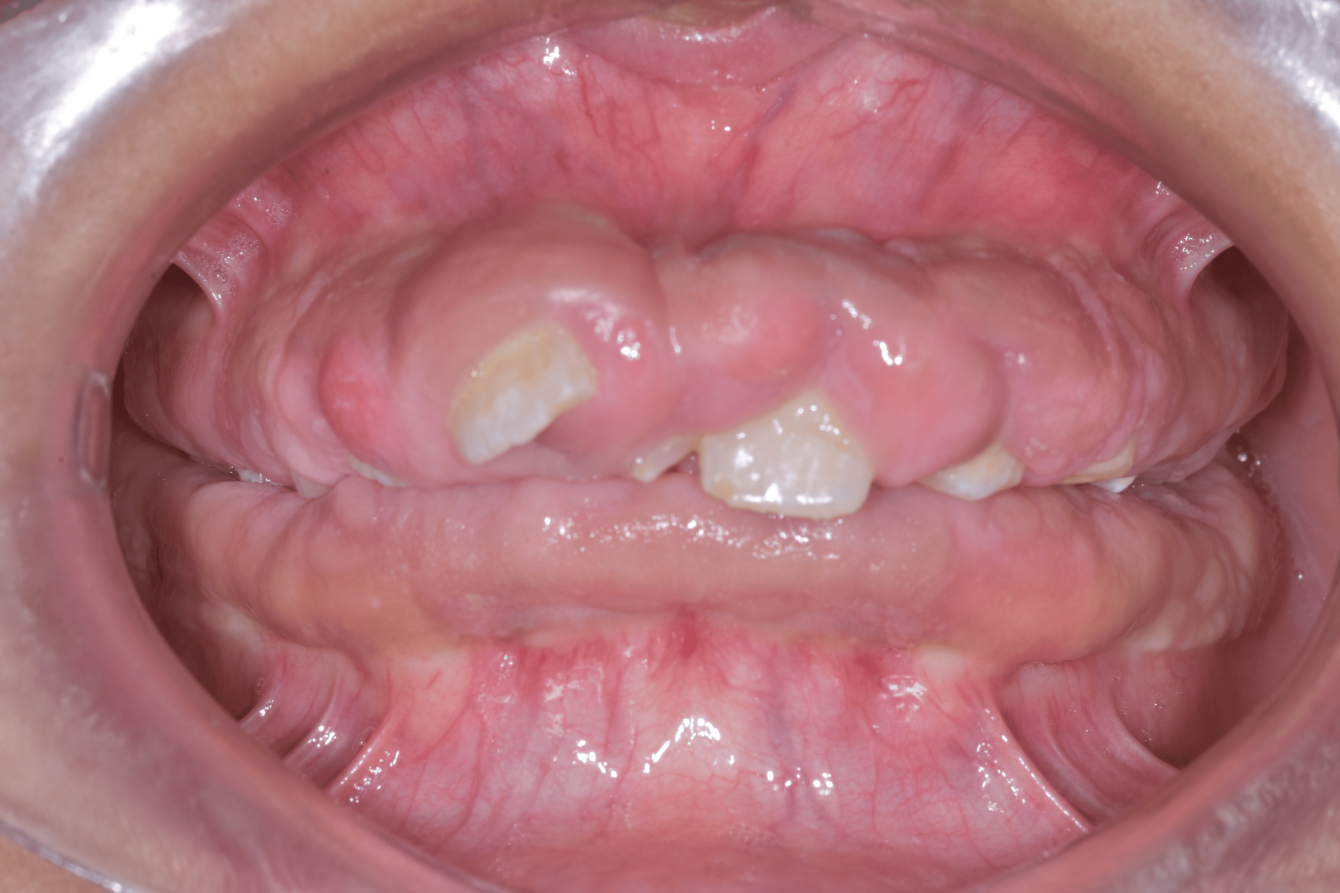
Intraoral view of gingival fibromatosis

An 11-year-old male patient presented to the periodontology department of the Faculty of Dental Medicine of Monastir in Tunisia with a chief complaint of progressive gingival enlargement. The condition had gradually increased in size, leading to significant functional and aesthetic concerns. The patient had no relevant medical history and was not taking any medications. The gingival enlargement was first noticed by the patient’s parents after the eruption period of the permanent teeth, as several teeth remained partially covered by gingival tissues, while others were completely retained. There was no reported family history of gingival overgrowth or similar oral conditions (Figures 1-2).

**Figure 3:**
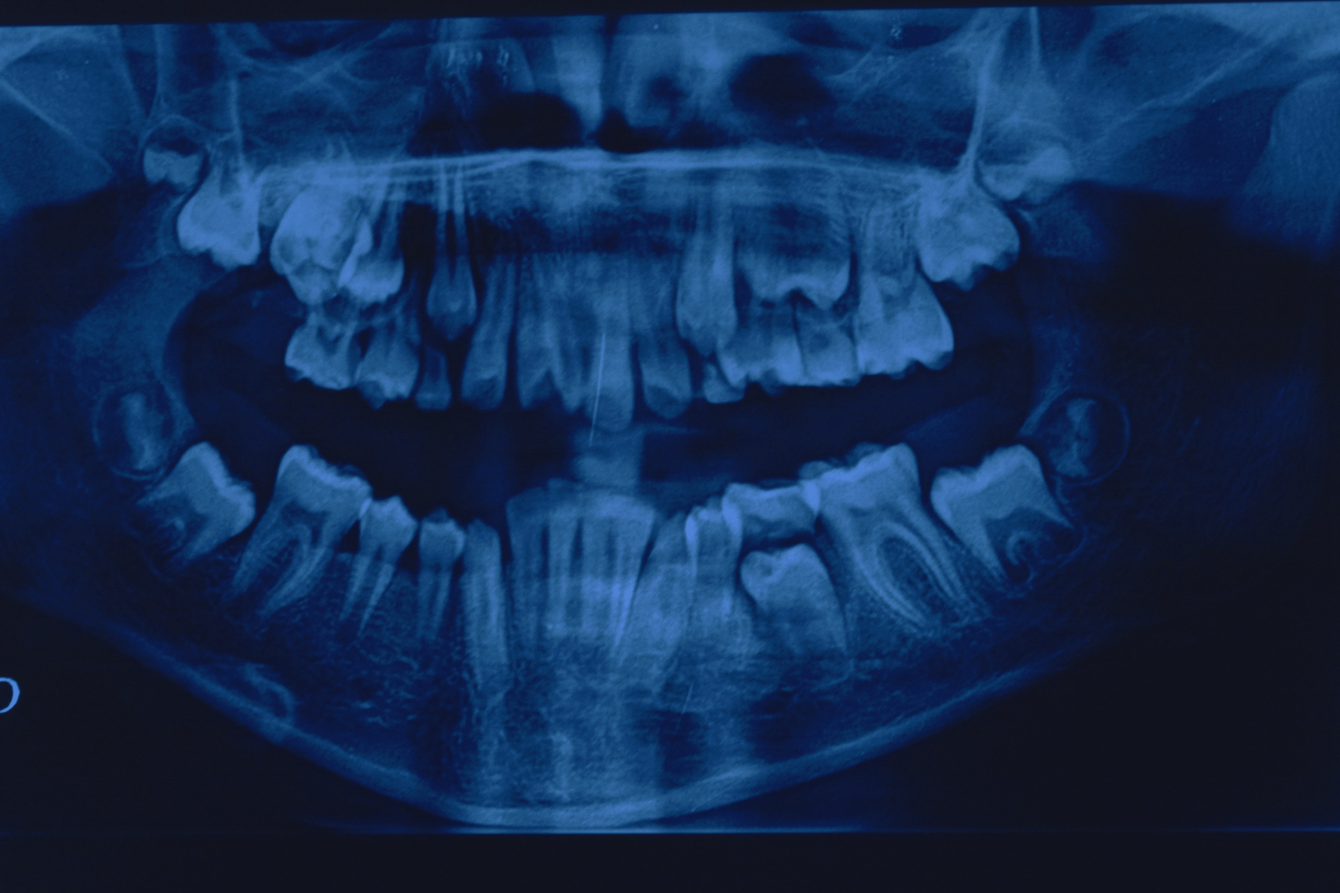
Panoramic radiography revealing multiple malpositions and retained teeth

Clinical examination revealed generalized gingival enlargement, involving both the maxillary and mandibular arches, covering at least half of the dental crowns and, in some areas, almost the entire crown surface, leading to the formation of deep gingival pseudopockets with probing depths of up to 12 mm, and most of them were bleeding on probing. A significant amount of plaque was observed, likely related to the patient’s challenging socioeconomic background and limited access to adequate oral hygiene resources. The gingival tissues appeared enlarged and fibrotic, partially covering the crowns of multiple teeth, while some teeth were almost fully covered. The patient demonstrated poor oral hygiene, likely related to the patient’s challenging socioeconomic background and limited access to adequate oral hygiene resources. The excessive gingival tissue resulted in functional impairment, including difficulty with mastication and speech. Panoramic radiography revealed multiple malpositions and retained teeth (Figure 3). Additionally, the patient exhibited mouth breathing and was unable to achieve complete lip closure due to the bulkiness of the gingival tissues.

**Figure 4:**
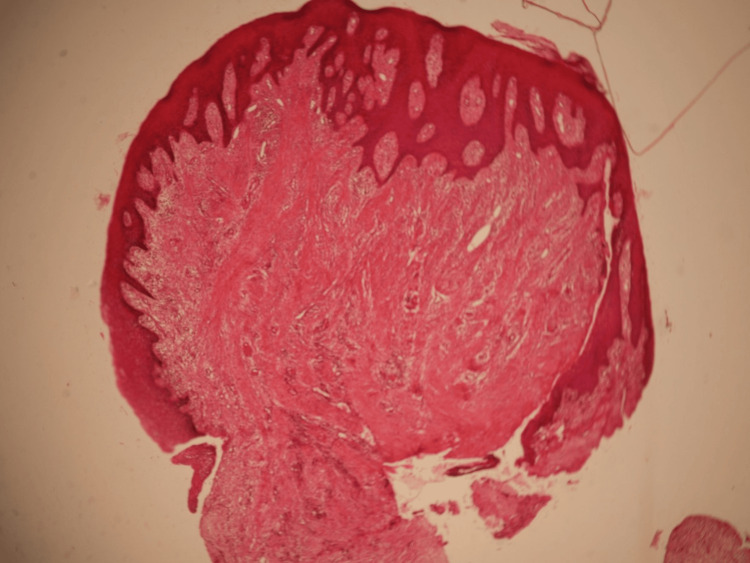
Histological slide of excised tissues

Laboratory investigations, including routine blood tests, were within normal limits, along with the pediatric consultation, helping to exclude systemic or hematologic causes. The family history and the absence of any medication excluded other forms of gingival overgrowth, namely hereditary gingival fibromatosis and drug-induced gingival overgrowth, respectively. Beyond the physical findings, the condition had a notable psychosocial impact. The patient reported social anxiety, reluctance to speak, and progressive social isolation, primarily due to aesthetic concerns and speech difficulties. The histological hematoxylin and eosin staining exam revealed the density of type 1 collagen fiber within the non-oriented connective tissue, along with an abundant inflammatory infiltrate, confirming the diagnosis of gingival fibromatosis (Figure 4).

**Figure 5:**
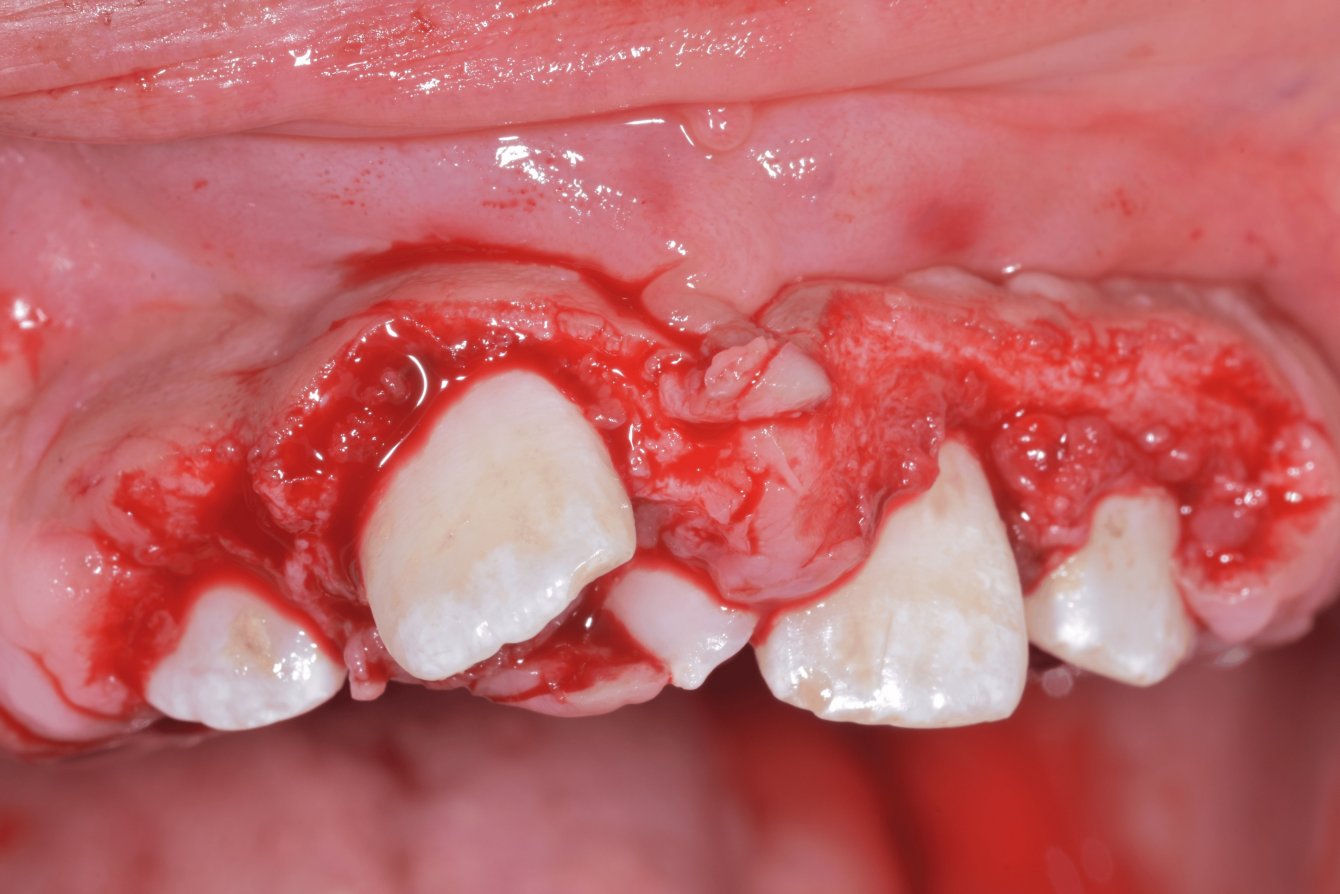
Maxillary gingivectomy incisions

**Figure 6:**
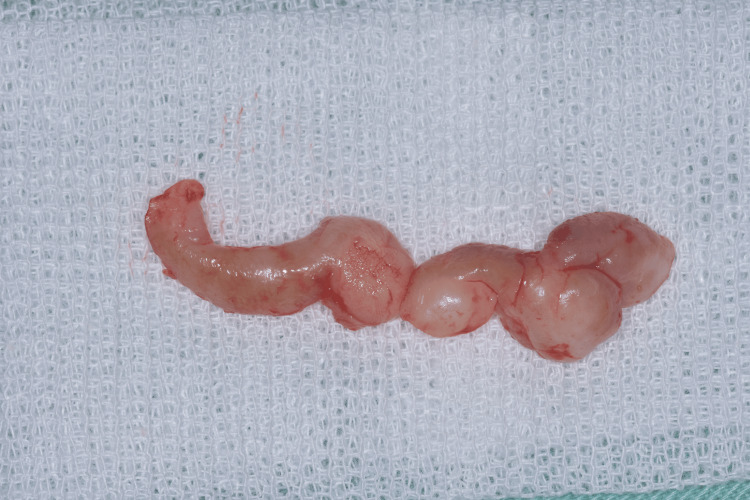
Excised fibrotic gingival tissues gingival tissues

**Figure 7:**
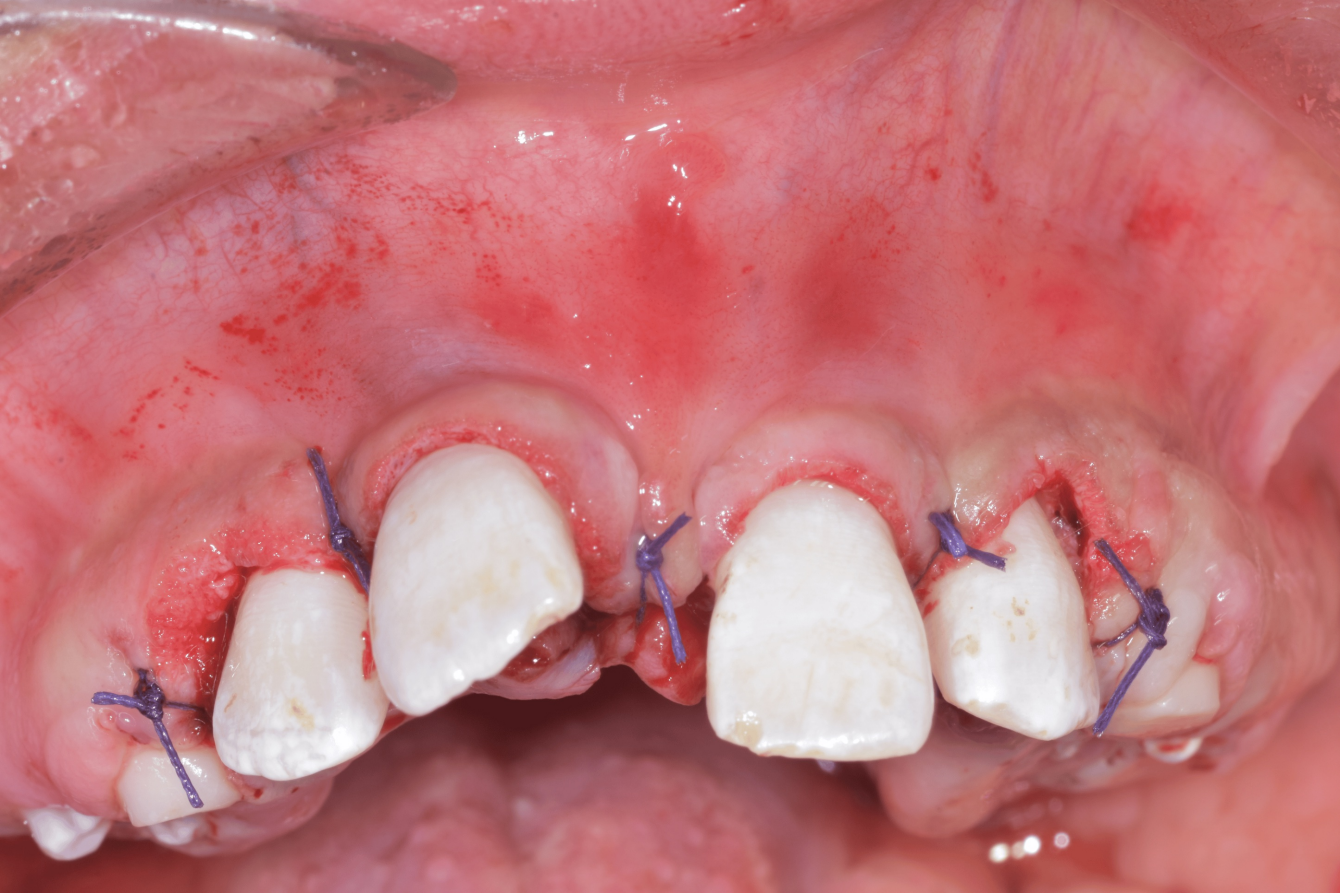
Maxillary gingivectomy sutures

Following completion of the initial periodontal therapy comprising oral hygiene improvement, plaque control measures, and scaling and root planing , surgical treatment was performed under local anesthesia in multiple sessions to ensure proper healing and patient comfort. Both external and internal bevel gingivectomy techniques were utilized to remove the excessive fibrotic gingival tissue and re-establish normal gingival contours. The external bevel gingivectomy was carried out using Kirkland and N°11 blades to excise the enlarged tissue and reduce gingival bulk, allowing improved crown exposure and facilitation of oral hygiene. This was followed by an internal bevel gingivectomy and interrupted O sutures (Figures 5-7).

**Figure 8:**
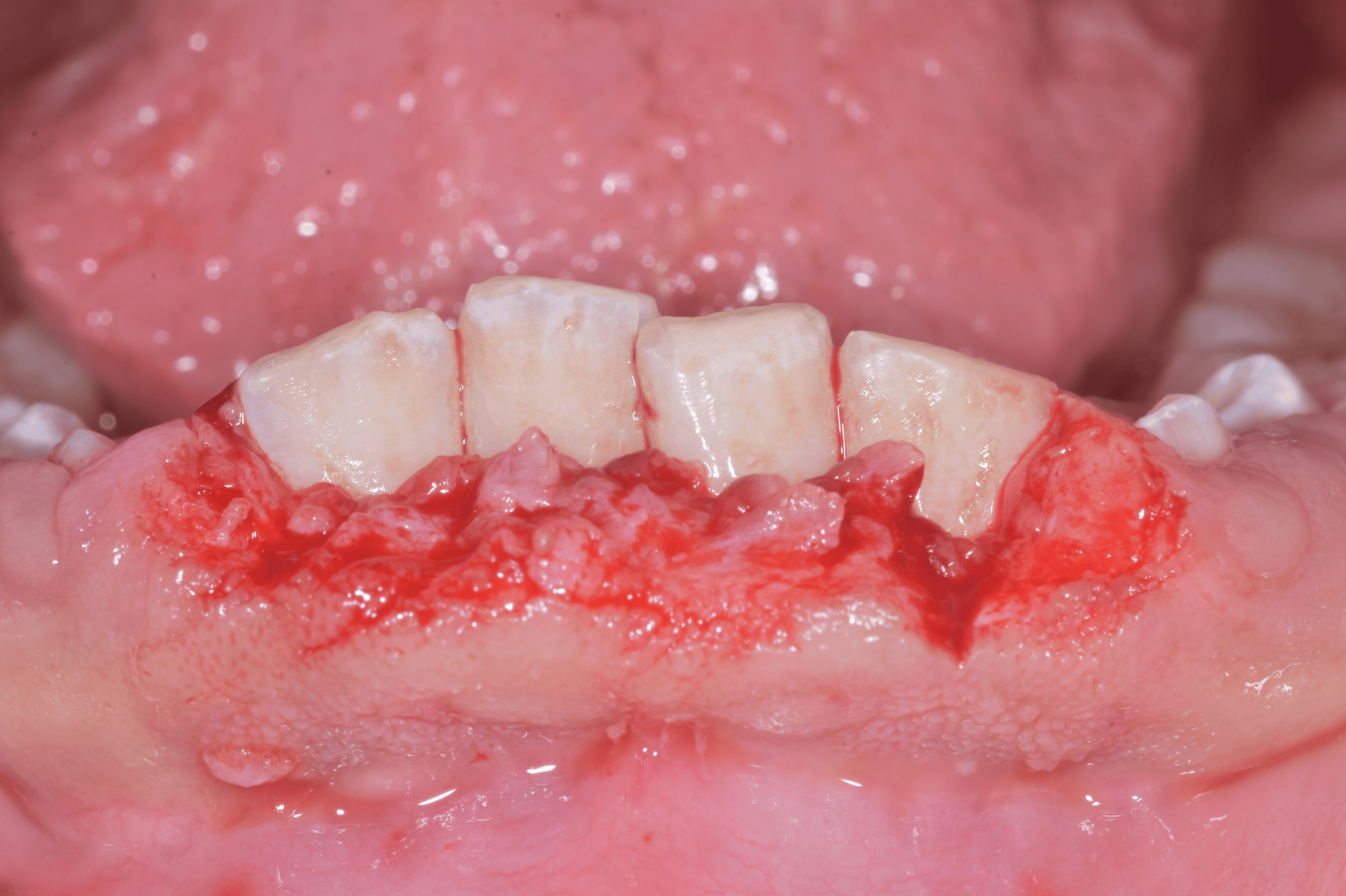
Mandibular gingivectomy incisions

**Figure 9:**
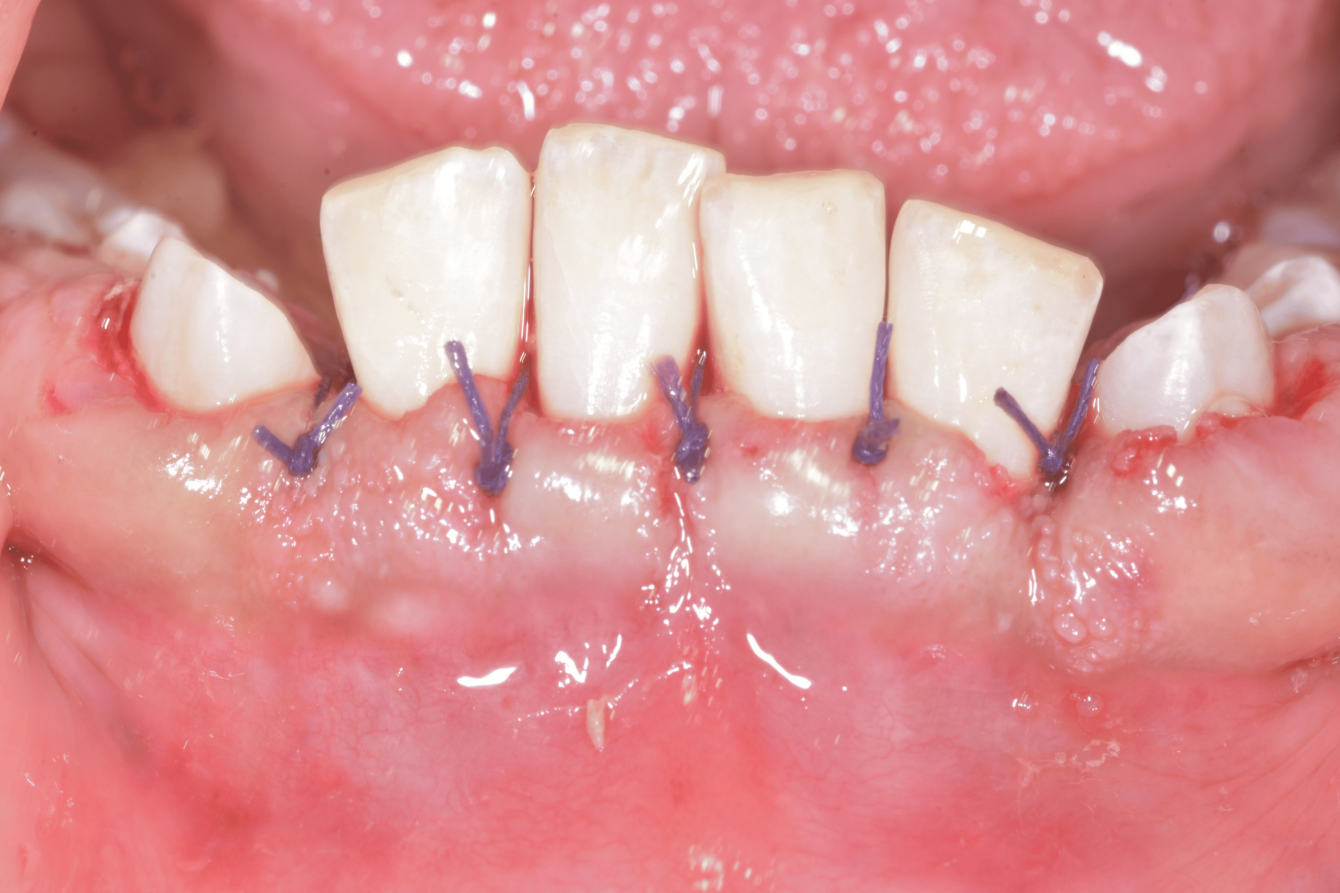
Mandibular gingivectomy sutures

The internal bevel technique involved making an incision from the inner aspect of the gingiva to elevate a mucoperiosteal flap. This allowed direct visualization of the underlying structures and completion of thorough scaling. Excess fibrotic tissue was trimmed from the internal surface of the reflected flap to reduce its thickness. The flap was then repositioned and sutured with single O sutures to achieve optimal contouring and improved gingival architecture (Figures 8-9). Additionally, following the gingivectomy, several retained temporary teeth were extracted to facilitate the eruption of permanent teeth.

**Figure 10:**
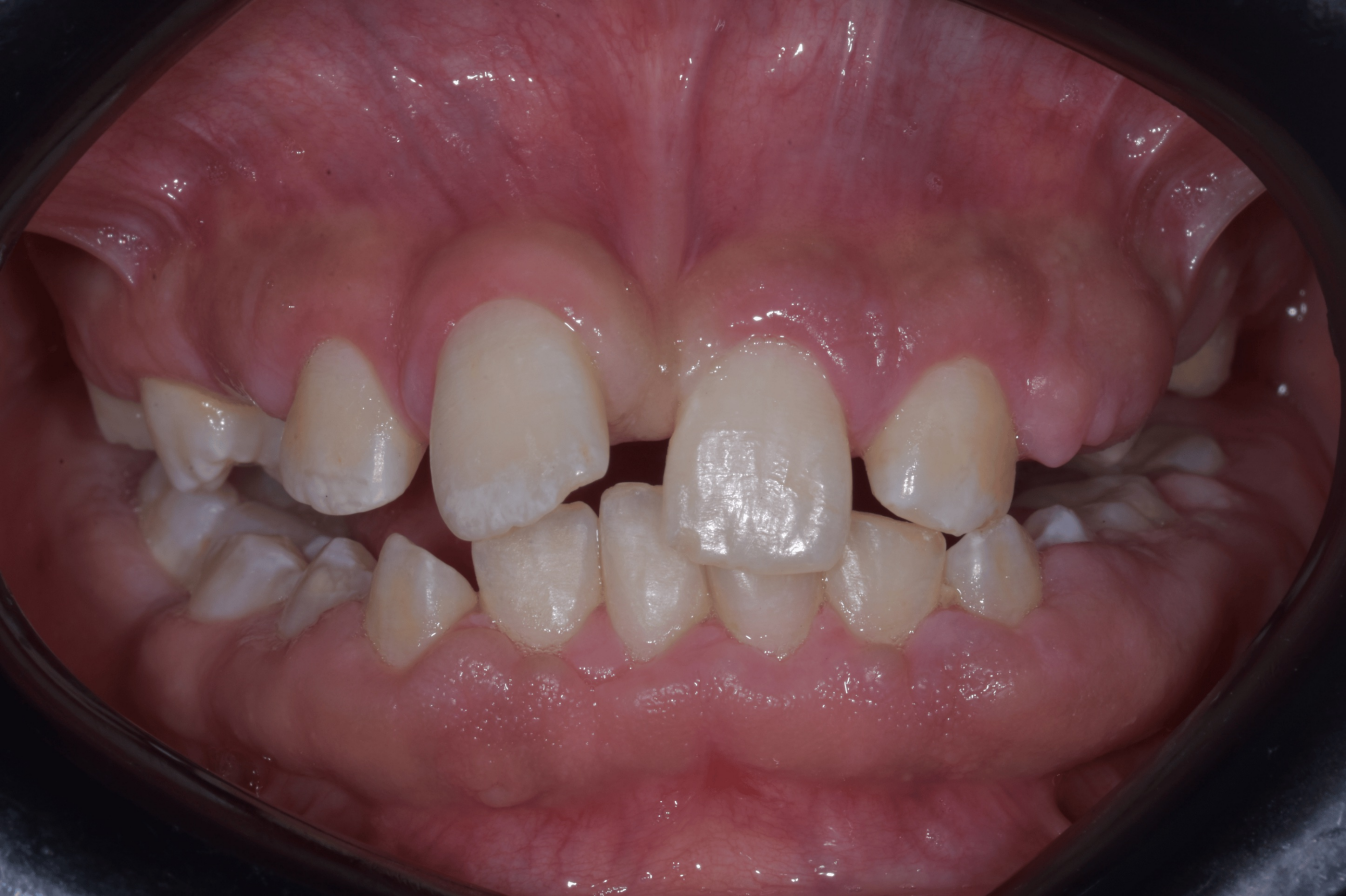
Six-month postoperative intraoral view

**Figure 11:**
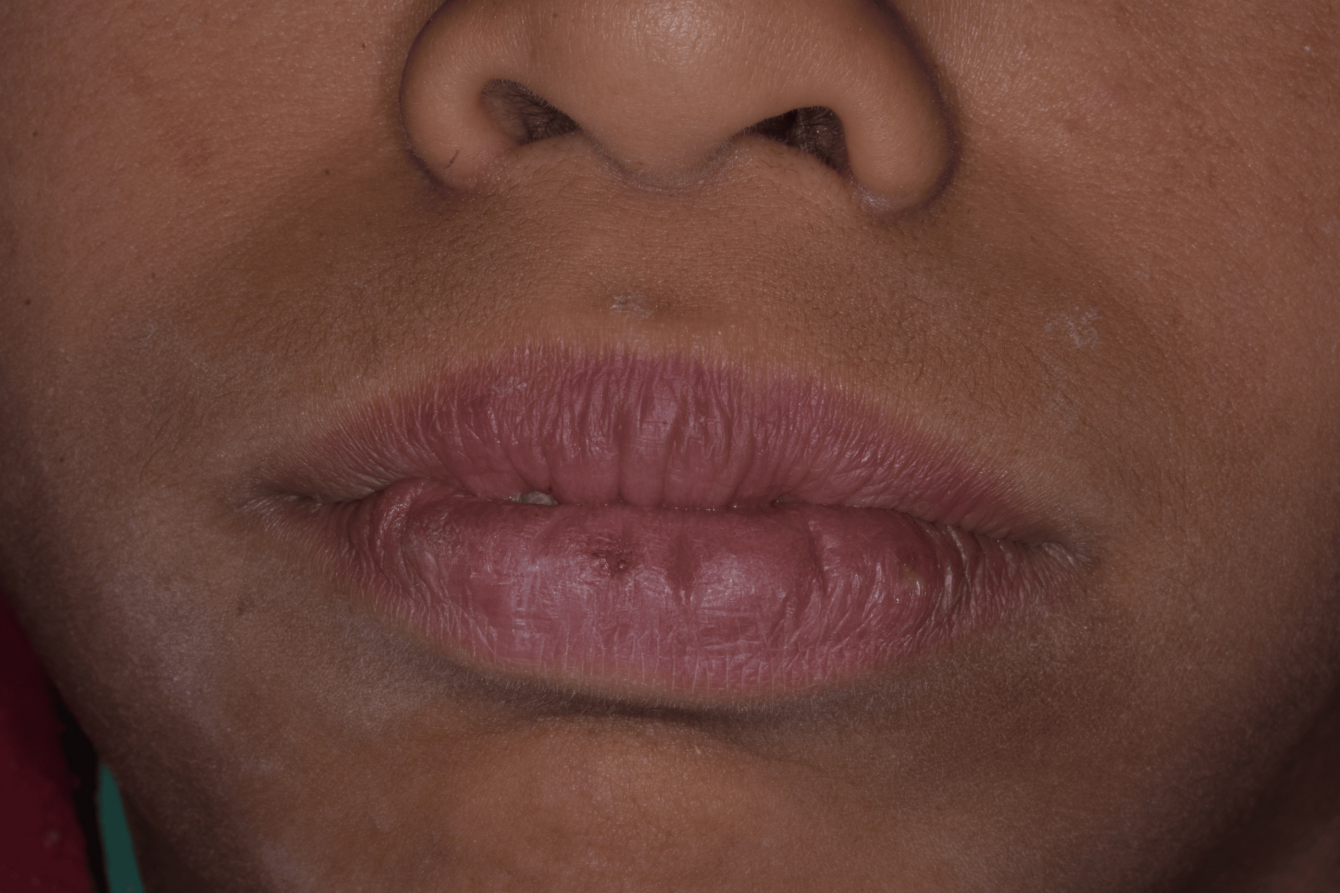
Six-month postoperative extraoral view of lip closure

Postoperative management included the prescription of 1.5 g of paracetamol per day for pain control and 0.12% chlorhexidine mouthwash three times a day for seven days. The patient was monitored regularly with biweekly visits followed by monthly visits after the surgical procedures to assess healing, functional improvements, and potential recurrence of gingival overgrowth. Oral hygiene instructions were reinforced at each visit, and plaque control was carefully evaluated to ensure optimal periodontal health. Six months after all surgeries were completed, the clinical results were highly favorable. The gingival contours remained stable, with no signs of relapse. The patient regained complete lip closure, and functional improvements were evident, including enhanced mastication, clearer speech, and resolution of mouth breathing. In addition, the psychosocial impact of the condition had markedly improved; the patient and his family reported increased self-confidence, reduced social anxiety, and greater willingness to engage in social interactions. The patient is presently awaiting orthodontic intervention to rectify residual malocclusion. Long-term follow-up will continue to monitor for recurrence and to support maintenance of oral hygiene and periodontal health (Figures 10-11).

## Discussion

Gingival overgrowth is defined as an abnormal increase in the size of the gingival tissues resulting from inflammatory, drug-induced, systemic, or genetic causes. Depending on its etiology, it may present as edematous enlargement associated with plaque accumulation or as a dense fibrotic expansion of the gingival connective tissue. Accurate diagnosis requires careful evaluation of clinical features, medical history, and histopathological findings.

Gingival fibromatosis is a rare form of non-inflammatory gingival enlargement characterized by progressive fibrous overgrowth of the gingiva due to excessive accumulation of collagen within the connective tissue. It may occur as a hereditary condition, often transmitted in an autosomal dominant pattern, or as an idiopathic form without identifiable genetic, systemic, or pharmacological causes. The present case report describes the idiopathic variant, in which no relevant medical history, medication use, or family history of gingival overgrowth was identified. Idiopathic gingival fibromatosis is considered an uncommon condition, with an estimated prevalence of approximately one in 750,000 individuals [1-3].

Clinically, IGF usually manifests during childhood or adolescence and may present as localized or generalized enlargement affecting both maxillary and mandibular gingiva. In the present case, the patient exhibited generalized fibrotic enlargement leading to impaired mastication, speech difficulties, mouth breathing, and inability to achieve lip competence. These findings are consistent with previously reported cases describing functional and aesthetic compromise in severe forms of the disease. Histopathologically, IGF is characterized by dense fibrous connective tissue containing abundant type I collagen bundles, relatively few blood vessels, and minimal inflammatory infiltrate. The findings in our patient were consistent with these characteristics, confirming the diagnosis.

The differential diagnosis of IGF is essential, as several conditions may present with similar gingival enlargement. Drug-induced gingival overgrowth, particularly associated with anticonvulsants, calcium channel blockers, and immunosuppressants, must be excluded through a thorough medical history assessment. Inflammatory gingival enlargement secondary to plaque accumulation should also be considered; however, such cases typically demonstrate pronounced inflammatory features rather than dense fibrotic tissue. Systemic conditions such as leukemia may present with gingival enlargement, often accompanied by hematologic abnormalities, which were not observed in this patient, as blood tests were within normal limits. Syndromic forms of gingival fibromatosis associated with conditions such as Zimmermann-Laband syndrome or other genetic disorders should also be ruled out through clinical examination and history. In this case, the absence of systemic manifestations or familial history supported the diagnosis of IGF [4-6].

Management of this case was further complicated by the patient’s young age and socioeconomic background. Being only 11 years old, he could not tolerate prolonged surgical sessions, necessitating multiple staged procedures. Additionally, coming from a disadvantaged family with limited parental education affected oral hygiene practices and required intensive motivation and support.

Surgical intervention remains the treatment of choice in moderate to severe cases. In this patient, both external and internal bevel techniques were employed to achieve adequate tissue reduction and optimal contouring. The use of internal bevel incisions with flap elevation allowed thorough scaling and reduction of fibrotic tissue thickness before apical repositioning, contributing to improved gingival architecture and facilitating oral hygiene maintenance [7]. Extraction of retained temporary teeth further supported the normal eruption of permanent dentition. It has been reported in the literature that some authors prefer electrosurgery in the management of gingival fibromatosis due to the dense fibrous consistency of the tissue, which can make excision with a scalpel more difficult, while laser techniques may produce a more superficial effect. Additionally, electrosurgery offers the advantage of a relatively bloodless surgical field; nevertheless, in cases presenting with extensive and dense fibrotic tissue, these methods may be less time-efficient for large tissue reduction when compared to conventional scalpel-based surgical techniques. [8].

Recurrence is a recognized challenge in IGF management and is often associated with poor plaque control or incomplete excision. At six months postoperatively, there were no signs of relapse observed in our patient, and functional improvements were significant. The patient regained lip competence, demonstrated improved mastication and speech, and experienced notable psychological benefits, including reduced social anxiety and increased self-confidence. The psychological impact was particularly profound. Prior to treatment, the patient exhibited social withdrawal, reluctance to speak or smile, and marked isolation. Following surgical correction, he demonstrated a remarkable boost in confidence, greater social engagement, and improved quality of life. Continued follow-up remains essential to monitor for potential recurrence and to support long-term periodontal stability, especially as the patient prepares for orthodontic treatment.

This case highlights the importance of early diagnosis, comprehensive differential assessment, and carefully planned surgical management in IGF. Reporting such cases contributes to a better understanding of this rare condition. Furthermore, it reinforces the need for multidisciplinary and long-term care.

## Conclusions

Idiopathic gingival fibromatosis remains a rare, benign entity causing profound functional (mastication, speech, delayed eruption) and psychosocial burdens, particularly in pediatric cases. Gold-standard diagnosis demands meticulous clinicopathologic correlation, i.e., demonstrating acellular fibrosis with minimal vascularity and excluding mimics based on history, imaging, and genetics. Surgical resection via wide bevel gingivectomy or lasers, phased by age and extent, constitutes definitive therapy; adjunctive plaque control (chlorhexidine, antimicrobials) and regular recalls mitigate high recurrence. This case exemplifies how staged, multidisciplinary intervention, even in socioeconomically challenged patients, yields durable aesthetic, functional, and psychological gains, underscoring imperatives for precocious detection and lifelong oversight in the IGF armamentarium.
